# Optimization of Hydroponic Wheat Sprouts as an Alternative Livestock Feed: Yield and Biochemical Composition Under Different Fertilization Regimes

**DOI:** 10.3390/plants14142166

**Published:** 2025-07-14

**Authors:** Andrius Grigas, Dainius Steponavičius, Indrė Bručienė, Ričardas Krikštolaitis, Tomas Krilavičius, Aušra Steponavičienė, Dainius Savickas

**Affiliations:** 1 Faculty of Engineering, Vytautas Magnus University Agriculture Academy, Studentų St. 15A, Akademija, LT-53362 Kaunas, Lithuania; dainius.steponavicius@vdu.lt (D.S.); indre.bruciene@vdu.lt (I.B.); dainius.savickas@vdu.lt (D.S.); 2 Faculty of Informatics, Vytautas Magnus University, LT-44248 Kaunas, Lithuania; ricardas.krikstolaitis@vdu.lt (R.K.); tomas.krilavicius@vdu.lt (T.K.); 3 Catering Department, Kaunas University of Applied Sciences, Pramonės Ave. 22, LT-50387 Kaunas, Lithuania; steponaviciene.ausra@gmail.com

**Keywords:** hydroponic forage production, precision livestock feeding, nutrient optimization, feed quality, alternative feed systems

## Abstract

This study investigated the effects of macronutrient type and concentration on the biomass yield and biochemical composition of hydroponically grown wheat sprouts (HWS), with the aim of identifying fertilization strategies that optimize both productivity and feed quality. HWS were cultivated using a nutrient film technique over a 7-day period under controlled environmental conditions, with treatments including calcium nitrate (CN1–CN3), potassium phosphate (CP1–CP3), potassium sulfate (CK1–CK2), and a balanced NPK 20–20–20 fertilizer (NPK1–NPK3), each applied at three increasing concentrations. The quantitative parameters assessed included biomass yield per unit of dry seed (DP, kg kg^−1^) and dry matter content (DM, %), while qualitative traits included crude protein (CP), ether extract (EE), crude fiber (CF), and ash content. Results indicated that balanced NPK fertilization significantly enhanced performance, with NPK3 achieving the highest biomass yield (6.39 kg kg^−1^), CP (24.26%), CF (5.63%), and ash (16.0%) content. In contrast, CN3 treatments reduced yield (4.84 kg kg^−1^) despite increasing CP (19.65%), indicating trade-offs between nitrogen enrichment and vegetative expansion. Phosphorus-based treatments (CP2–CP3) improved nutrient density without suppressing yield. Regression analyses revealed strong correlations between DM and both CF (R^2^ = 0.81) and ash (R^2^ = 0.71), supporting their utility as indirect indicators of feed quality. EE content remained stable (2.07–2.67%) across all treatments, suggesting its limited responsiveness to macronutrient manipulation. These findings highlight the importance of nutrient synergy in hydroponic systems and provide a practical framework for tailoring fertilization regimes to meet specific agronomic and nutritional objectives in precision livestock feeding and provide practical guidance for optimizing hydroponic livestock feed production.

## 1. Introduction

Feeding the world’s growing population is becoming an increasingly complex challenge. As demand for meat, dairy, and other animal products rises, pressure increases on agricultural systems to deliver not just more feed, but feed that is sustainable, nutritious, and produced with fewer natural resources. Traditional forage crops, such as alfalfa and maize silage, continue to play a major role in livestock diets, but their reliance on fertile land, intensive water use, and seasonal conditions makes them vulnerable to supply fluctuations and environmental concerns [1,2]. At the same time, the carbon footprint associated with large-scale forage production remains a significant issue [3], and as climate change accelerates, agriculture must adapt [4].

One promising approach that has gained attention in recent years is hydroponic cultivation [5]. Unlike conventional farming, hydroponics allows plants to grow without soil, offering a controlled environment where water and nutrient use can be carefully managed. Studies show hydroponic systems can decrease water usage by up to 90% while drastically reducing land requirements [6,7]. This technology enables efficient fodder production in environments where conventional agriculture is constrained—such as arid regions with limited water availability or space-restricted settings like peri-urban or controlled environment systems [8,9].

Among the crops suited for hydroponic production, wheat sprouts (HWS) stand out. They grow quickly, reaching harvest in just 7 to 10 days, and offer a dense package of crude protein, minerals, antioxidants, and bioactive compounds [10,11]. Their nutritional profile, combined with high digestibility, has made HWS a strong candidate as an alternative forage, particularly for ruminants. Research also points to HWS improving rumen efficiency and reducing methane emissions, adding environmental benefits to their list of advantages [12,13].

However, in hydroponic systems, plants are entirely dependent on the nutrient solution supplied for their growth and development. This makes the composition of that solution critical—not only for maximizing biomass but also for tailoring the nutritional qualities of the final product. Key macronutrients like nitrogen (N), phosphorus (P), and potassium (K) are fundamental to plant function. N is vital for protein and amino acid synthesis; P is involved in energy metabolism and structural integrity; and K supports enzyme activity, osmotic regulation, and stress tolerance [14,15].

Despite these well-known roles, relatively little research has been performed to explore how varying the balance and concentration of N, P, and K specifically affects both the yield and the feed quality of HWS. Most previous studies have looked either at biomass alone or at the effect of individual nutrients, rather than considering their interactions [16,17]. In practical animal nutrition, the value of a feed source is determined not solely by its biomass yield, but also by its internal composition, specifically the concentrations of crude protein, fiber, minerals, and its overall digestibility. Also, future studies will include detailed mineral and ionic composition analysis to better assess feed quality.

An additional complexity arises from the fact that the augmentation of a single nutrient can result in trade-offs, potentially enhancing certain plant traits while adversely affecting others. For instance, adding more nitrogen might boost protein content, but could also suppress lipid accumulation. Similarly, an excess of potassium can raise fiber content, which might hurt digestibility if it goes too far [18,19]. These interactions mean that simply maximizing one nutrient will not necessarily produce the best overall feed.

Considering this, the current study was undertaken to evaluate how different fertilization regimes, applying nitrogen, phosphorus, and potassium alone and in combination, influence the biomass production and biochemical composition of HWS. It was hypothesized that both the type and concentration of macronutrient fertilizers would have a significant impact on the quantitative yield and composite quality of HWS. Furthermore, the study investigated whether the relationships between nutrient concentrations and plant responses exhibited linear or non-linear trends, with the objective of identifying threshold levels beyond which additional fertilizer inputs fail to yield significant benefits and may even negatively impact feed quality.

The main hypothesis of this study was that both the type and concentration of macronutrient fertilizers would have a significant impact on the quantitative yield and composite quality of HWS. It was also aimed to identify and demonstrate that targeted nutrient modulation can be used to direct plant development towards desirable traits, such as higher protein, fiber content, or increased yield. These traits are particularly important in precision agriculture and livestock systems, which require both efficiency and nutritional adequacy. By addressing these questions, this research seeks to support the development of nutritionally optimized hydroponic feed systems that align with both economic and environmental goals in modern livestock production.

## 2. Materials and Methods

The studies were conducted in 2023–2025 at the Agricultural Machines Technological Process Research Laboratory of Vytautas Magnus University Agriculture Academy, Lithuania.

### 2.1. Hydroponic System Setup

**Figure 1:**
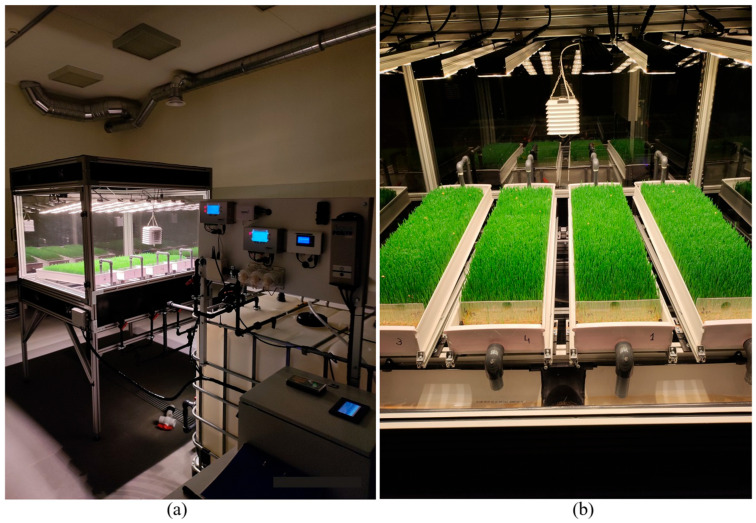
(**a**) HWS cultivation stand HAS1 and (**b**) HWS during cultivation.

Scientific research was carried out using specialized equipment adapted to hydroponic cultivation and scientific research—HWS cultivation stand HAS1 (Figure 1).

**Figure 2:**
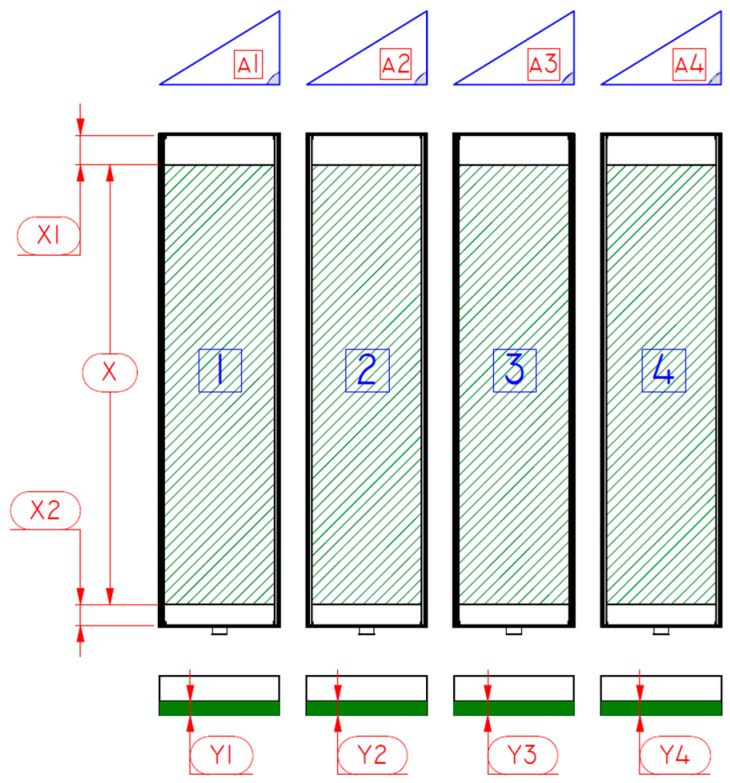
Growth trays and seed spreading parameters in the HAS1 stand. A1–A4—slope angle α (%), Y1–Y4—thickness of spread seeds (mm), X—length of spread seeds (mm), X1 and X2—distance from the edges of the growth trays (mm).

Its structure (2150 × 1400 × 1400 mm) was constructed from extruded aluminum profiles. The interior of the bench houses a unit for HWS cultivation and measurement, featuring four growth trays (Figure 2), each sized 1000 × 225 × 70 mm. Each tray offers a growing space of 0.9 m^2^. The nutrient film technique (NFT) was chosen as the nutrient solution supply system. The core mechanism of the NFT involves technologically inclining the growth trays at a predetermined angle, denoted as α, to facilitate the delivery of the nutrient solution to the plants. This configuration leverages the disparity in potential energy to induce a gravitational flow of the nutrient solution across the plant roots arrayed along the inclined plane. This ensures an efficient distribution of humidity, oxygen, and nutrients essential for plant growth. Subsequently, the solution flows to the lowest part of the shelf, where it is either directed towards waste management systems or subjected to purification and recycling processes for further use. In this empirical study, the nutrient solution that passed through the roots was discharged into the sewer and not reused to reduce as much as possible the growth of various pathogens and the imbalance of nutrients. This hydroponic cultivation methodology is extensively utilized for a wide spectrum of agricultural crops, demonstrating pronounced effectiveness for species necessitating shorter growth periods, such as lettuce and various leafy vegetables [20]. The NFT system is flexible and beneficial for the environment, making it great for innovative farming methods. It helps save water and uses nutrients wisely, promoting water conservation and nutrient efficiency.

The slope angle α of the HAS1 stand growth trays was set at 6.5% [21]. Other parameters indicated in Figure 2 were chosen as follows: the thickness of the spread seeds Y1–Y4—30 mm, the length X—825 mm, and the distances from the edges of the growth trays X1 and X2—85 mm each (Figure 2).

### 2.2. Hydroponic Wheat Sprouts Growth Parameters

To accurately assess the effects of different fertilization strategies on HWS, it is essential to establish a controlled and standardized growth environment. The following section outlines the key cultivation parameters maintained during the experiment, ensuring reproducibility and minimizing external variability that could influence biomass yield or biochemical composition.

#### 2.2.1. Microclimate

The temperature throughout the growth cycle was dependent on the temperature of the laboratory room containing the HAS1 stand. The relative humidity of the environment in the growth chamber was controlled (<60%) with an installed air extraction and intake system and monitored. The concentration of CO_2_ in the HAS1 stand was continuously monitored throughout the growth cycle to observe plant respiratory activity. No external CO_2_ supplementation was applied, and ambient levels were maintained without artificial control. This allowed the study to capture natural variations in CO_2_ concentration resulting from plant metabolism. All microclimatic parameters are presented in Table 1 that presents the average environmental parameters recorded during the growth cycle for each treatment, including temperature, relative humidity, CO_2_ concentration, and vapor pressure deficit (VPD), all of which remained within controlled ranges, with slight variations due to treatment-specific irrigation activity and plant transpiration rates.

#### 2.2.2. Lighting, Photoperiod, and Irrigation Strategy

The HWS growing stand HAS1 is equipped with a smart light emitting diode (LED) plant lighting system, which can create up to 1500 (μmol m^−2^) s^–1^ of photosynthetically active radiation flux density (PAR), which can be adjusted, as well as changing the light spectrum and duration (photoperiod). The intensity and color spectrum of the light could be modified to accommodate the specific needs of a plant variety, its stage of development, and desired growth outcomes. LEDs offer notable benefits over conventional plant growth lighting options, such as fluorescent lights, including higher efficiency in turning electricity into usable light and emitting less heat [22]. Since this study did not analyze various recipes of the light spectrum and, in general, the influence of light on the cultivation of HWS, a full spectrum (380–780 nm) light was chosen. Regarding the PAR and lighting duration, a light flux of 250 (μmol m^−2^) s^–1^ and a 12 h photoperiod were selected (from 8:00 a.m. till 8:00 p.m.).

Regarding the irrigation of HWS, a flow rate of 2 L min^–1^ of the nutrient solution was chosen so that the HWS and seeds receive a sufficient amount of water, nutrients, and oxygen, but at the same time, due to the high flow rate, especially in the first days of growth, the seeds would not be washed to the lowest part of the growth tray. Since the supply of nutrient solution was in all cases 8 times day^–1^ and irrigated for 60 s, the total amount of water per growth tray day^–1^ was 16 L.

#### 2.2.3. Preparation of Seeds and Cultivation of Hydroponic Wheat Sprouts

Spring wheat (*Triticum aestivum* L.) seeds of the Scirocco variety were used for an empirical study. Prior to testing, seed germination was determined using the moistened tissue method [23], and it was 98.1 ± 2.2%. Before starting each growth period, it is important to clean the seeds and the HAS1 stand to reduce the spread of diseases as much as possible. Sodium hypochlorite (NaOCl) solution was used to disinfect both the seeds and the surfaces of the HAS1 system. For seed disinfection, a 2% NaOCl solution was used, with seeds soaked for 10 min. For surface disinfection of the HAS1 system, a stronger 20% NaOCl solution was applied with a shortened exposure time of 5 min. After disinfection, the seeds and equipment surfaces were thoroughly rinsed with clean water to remove all the disinfectants. Disinfection of the seeds was followed by their soaking phase. During the seed soaking process, the seeds were immersed in tap water for a period of 24 h, allowing them to absorb moisture and begin the germination process. This procedure activates enzymes in the seeds that break down complex nutrients into simpler forms that are more easily absorbed by plants. In addition, soaking helps to soften the seed coat, promoting faster and more uniform germination. It also helps identify which seeds are likely to germinate because healthy seeds sink in water, while bad ones tend to float. Soaking ensures all seeds begin sprouting at the same time, leading to even growth.

After disinfection and soaking, the wheat seeds were sown manually onto each growth tray. A total of 1.55 kg of soaked seeds, equivalent to 1.00 kg of dry seeds, was used per tray. This corresponds to a seeding density of approximately 1.72 kg/m^2^ (dry seed basis), given each tray’s growing area of 0.9 m^2^. The soaked seed mass was determined based on pre-trial water absorption tests and standardized to ensure consistent germination and sprouting. The seed layer thickness was approximately 30 mm, as defined by the cultivation protocol. This seeding rate allowed uniform distribution and optimal conditions for hydroponic sprout development.

#### 2.2.4. Nutrient Solution

To evaluate the influence of different fertilizer types and concentrations on the quantitative and qualitative characteristics of HWS, a series of nutrient solutions were prepared using four common fertilizers: calcium nitrate (Ca(NO_3_)_2_), potassium phosphate (KH_2_PO_4_), potassium sulfate (K_2_SO_4_), and a balanced NPK 20-20-20 compound fertilizer. These compounds were selected based on their widespread use in hydroponic nutrient formulations, including Hoagland-type solutions, to isolate and compare the effects of nitrogen, phosphorus, potassium, calcium, and sulfur on plant development. Each fertilizer was tested at three concentration levels, coded as CN1–CN3, CP1–CP3, CK1–CK3, and NPK1–NPK3, respectively. These concentrations were selected based on published ranges used in hydroponic fodder cultivation and our preliminary optimization trials.

The nutrient solutions were prepared using 800 L of tap water per growth cycle. Prior to fertilizer addition, the physicochemical properties of the water were assessed to ensure compatibility with hydroponic systems. Tap water had a pH range of 7.3–7.7 and an electrical conductivity of 0.643 ± 0.015 µS/cm (Table 2). Key ions included chloride (16–18 mg/L), sulfate (24.9 mg/L), and sodium (11.0 mg/L), while nitrate and ammonium levels were below quantification limits. Although deionized water is recommended for controlled nutrition studies, untreated tap water was used to reflect practical, real-world hydroponic conditions. The low baseline ion concentrations were accounted for in the treatment design. These values complied with EU drinking water standards and did not necessitate pre-treatment. However, to optimize nutrient availability in the hydroponic environment, the pH of the water was adjusted from its natural range to 5.8–6.0 using nitric acid before the preparation of nutrient solutions. Fresh nutrient solutions were prepared prior to each growth cycle to prevent nutrient degradation or cross-contamination between treatments.

The calcium nitrate treatments (CN1–CN3) supplied nitrogen in the form of nitrate (NO_3_^−^) alongside calcium (Ca^2+^), with concentrations ranging from 100 to 300 mg L^−1^ of nitrogen and 143 to 429 mg L^−1^ of calcium. The potassium phosphate treatments (CP1–CP3) delivered phosphorus (H_2_PO_4_^−^) and potassium (K^+^) in concentrations ranging from 100 to 300 mg L^−1^ of phosphorus, corresponding to 126 to 378 mg L^−1^ of potassium. Potassium sulfate treatments (CK1–CK3) provided potassium and sulfur (SO_4_^2−^), with potassium ranging from 100 to 300 mg L^−1^ and sulfur from 18 to 54 mg L^−1^. Lastly, the NPK compound fertilizer treatments (NPK1–NPK3) included a mixture of nitrogen forms (NO_3_^−^, NH_4_^+^, and NH_2_), phosphorus, and potassium, at respective concentrations of 100 to 300 mg L^−1^ nitrogen, 43.6 to 130.8 mg L^−1^ phosphorus, and 83 to 249 mg L^−1^ potassium. The pH of the nutrient solution was adjusted and maintained between 5.8 and 6.2 throughout the growing cycle to ensure optimal nutrient availability.

#### 2.2.5. Investigated Quantitative and Qualitative Parameters

Quantitative and qualitative parameters were observed and recorded in this study. As for the quantitative parameters, the main ones were the yield potential of germinated seeds DP (kg kg^–1^) (i.e., the yield from one kilogram of dry seeds) and the proportion of dry matter (LST ISO 712:2010 [24]) in the feed (%) after a 7-day growth cycle. The DP was recorded using a Flintec PB7.5 (C3 accuracy (0.023%) class) load cells (Flintec Inc., Hudson, MA, USA). The sensor’s signals were amplified using Laumas TLB485 digital-analog weight transmitter (24-bit analog to digital converter, 4.8 kHz) (Laumas, Parma, Italy).

The following criteria were used to evaluate the qualitative parameters of HWS: crude protein (%)—ISO 20483:2006 [25], crude fiber (%)—ISO 6865:2000 [26], ash (%)—ISO 5984:2022(E) [27], ether extract content (%)—AOAC 920.39-1920/LST ISO 1443 [28].

### 2.3. Statistical Analysis

Each treatment was conducted with four replicates (*n* = 4) in a completely randomized design (CRD), with tray positions randomly assigned within the growth chamber to minimize environmental variability, and the duration of each growth cycle was 7 days. Samples were collected at the end of each cycle between 8:00 and 9:00 a.m. to minimize diurnal variation. Data was analyzed using analysis of variance (ANOVA) with Statistica 10.0 statistical software. The post hoc test for the significant difference (R_0.05_) was used to compare the arithmetic means of the data. R_0.05_ was calculated with a 95% level of confidence [29].

## 3. Results and Discussion

The results revealed that nutrient composition, not just concentration, was the main determinant of productivity and compositional outcomes. High levels of nitrogen or calcium did not necessarily improve biomass yield, and in some cases led to physiological stress or suboptimal resource allocation. These findings highlight the importance of balanced fertilization strategies and highlight the risks of overly simplistic nutrient supplementation, especially in closed-loop hydroponic systems where the system’s ability to resist changes in nutrient concentration, pH, or ionic balance when external inputs are added is limited.

Although HWS cultivation has been widely explored in recent years, few studies have simultaneously evaluated such a broad spectrum of physiological and biochemical parameters under controlled conditions.

### 3.1. Biomass Yield and Dry Matter Content Response to Fertilization

After extensive experimental studies, it became evident that the nutrient composition played a pivotal role in shaping both biomass yield and dry matter content. The control treatment (tap water), which received no supplemental macronutrients, produced 5.18 ± 0.22 kg kg^–1^ DP, with a DM content of 13.30%. The relatively high biomass yield in the control group can be explained using endogenous seed reserves, which support initial sprout development even in the absence of external macronutrient supplementation. This baseline allowed for comparative evaluation of the various fertilization regimes.

**Figure 3:**
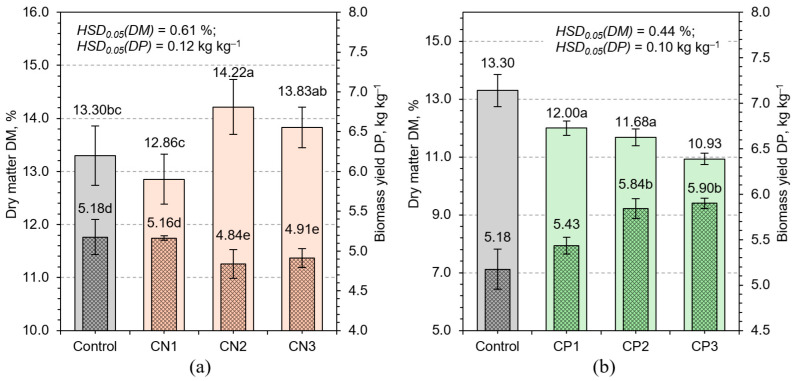
Effect of different calcium nitrate (**a**) and potassium phosphate (**b**) concentrations on biomass yield DP and dry matter content DM of HWS. Any two samples with a common letter are not significantly different (*p* < 0.05), as assessed using the least significant difference.

Among the tested treatments, calcium nitrate application revealed a distinct non-linear effect on growth. While the lowest concentration (CN1) slightly enhanced biomass production, higher concentrations (CN2 and CN3) led to significant reductions, accompanied by elevated DM content (Figure 3). This pattern suggests that moderate nitrate levels support vegetative expansion [30], but excessive concentrations may impose osmotic or oxidative stress, potentially due to nitrate accumulation and the ATP-intensive demands of nitrate reduction [31]. Importantly, this growth reduction is more plausibly attributed to nitrate-specific stress—including osmotic load and energy demand—rather than to a general nutrient deficiency. In this short-cycle hydroponic system, the seedlings rely primarily on internal seed reserves, and the solution was purposefully unbalanced to isolate the effects of individual macronutrients. Thus, the observed effects reflect excess nitrate, not the absence of other nutrients, and demonstrate the limits of applying Liebig’s Law in such early growth contexts. In addition, increased calcium availability may inhibit cell elongation by reinforcing pectin cross-linking within the cell wall matrix [32], thereby limiting volumetric growth despite higher tissue density.

In contrast, treatments with potassium phosphate (CP1–CP3) consistently promoted biomass accumulation across all tested concentrations, while maintaining relatively stable DM percentages (Figure 3). This uniform increase implies that biomass gains stemmed from effective tissue accumulation rather than water-driven expansion [33]. The synergistic effects of phosphorus, essential for ATP synthesis and nucleic acid formation, osmotic regulation, and enzymatic activation, led to sustainable and efficient biomass growth [34].

Potassium sulfate (CK1–CK3) treatments presented a more moderate response. Biomass increased by up to 8.3% under CK2, with minimal change in DM%, indicating a subtler but positive metabolic enhancement. Sulfur, incorporated into essential amino acids such as methionine and cysteine, contributes to protein synthesis and cellular function, while potassium continues to support energy-efficient carbon assimilation [35]. Although less dramatic than NPK or phosphate treatments, these findings highlight how potassium and sulfur can fine-tune metabolic efficiency, especially under specific environmental or developmental conditions.

**Figure 4:**
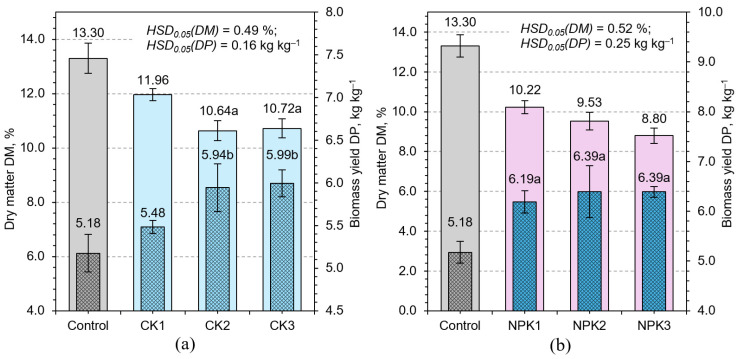
Effect of different potassium sulfate (**a**) and NPK 20–20–20 (**b**) concentrations on biomass yield, DP, and dry matter content, DM, of HWS. Any two samples with a common letter are not significantly different (*p* < 0.05), as assessed using the least significant difference.

Notably, the most pronounced increases in DP were recorded under NPK fertilization. NPK2 and NPK3 treatments enhanced biomass by 16.0% and 19.7%, respectively, with only modest increases in DM% (Figure 4). These results underscore the critical role of nutrient synergy, where simultaneous provision of nitrogen, phosphorus, and potassium supports both expansive growth and compositional integrity. The observed results likely reflect coordinated physiological processes such as increased nutrient uptake, hormonal balance, and synchronized cell division and elongation, culminating in high productivity without the typical trade-offs associated with dilution effects.

**Figure 5:**
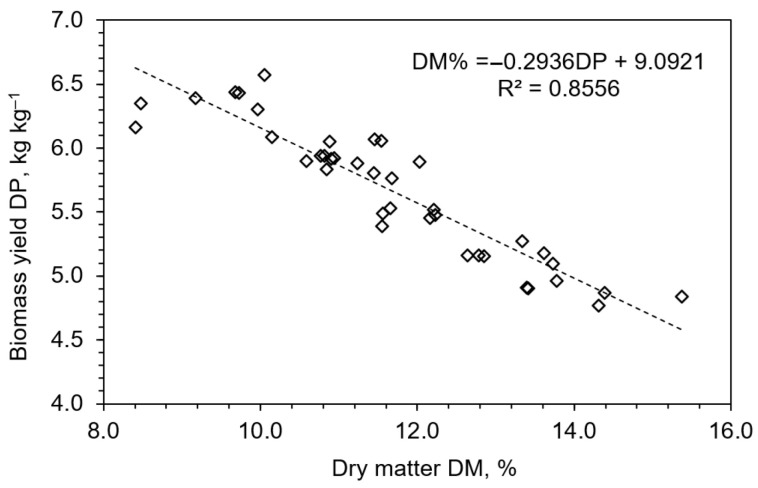
Linear regression between dry matter content (DM, %) and biomass yield (DP, kg kg^−1^) across all fertilizer treatments.

Regression analysis further highlighted these dynamics. A strong inverse correlation between biomass yield and DM% (R^2^ = 0.86) was observed (Figure 5), aligning with the well-established dilution effect in rapidly expanding tissues. NPK treatments deviated from this trend, suggesting that balanced nutrition can decouple this relationship to some extent, enabling robust growth without compromising tissue density. Treatments that induced physiological stress, such as CN3, exhibited reduced biomass yet elevated DM%, likely reflecting increased osmolyte or structural carbohydrate accumulation (Figure 3).

These physiological insights hold practical significance for feed production systems. High-yield scenarios appear best supported by balanced fertilization strategies, particularly those incorporating complete NPK blends (e.g., NPK3). However, treatments such as CP2 and CK2 may offer strategic advantages when the production goal shifts toward improving nutrient density or digestibility per unit mass. Thus, the choice of fertilization regime can be tailored to align with distinct agronomic or nutritional objectives. Importantly, the observed trade-offs reinforce the value of adaptive nutrient management practices that integrate ongoing compositional monitoring to optimize both productivity and feed quality.

### 3.2. Effect of Fertilization on Feed Quality Parameters

Beyond DP (kg kg^–1^) and DM (%), the nutritional value of HWS is defined by its compositional profile. This empirical study focused on four primary feed quality metrics: crude protein (CP), ether extract (EE), crude fiber (CF), and ash. Each of these parameters responds differently to nutrient availability, reflecting distinct metabolic and developmental pathways. Understanding their response to fertilization is crucial for designing nutrient strategies that optimize both growth and feed quality.

#### 3.2.1. Crude Protein and Ether Extract

**Figure 6:**
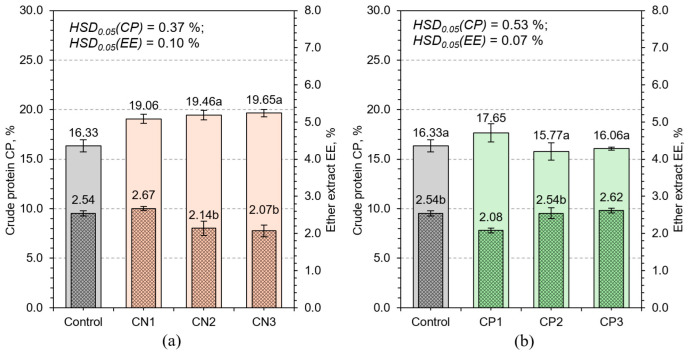
Effects of different calcium nitrate (**a**) and potassium phosphate (**b**) concentrations on crude protein CP and ether extract EE content of HWS. Any two samples with a common letter are not significantly different (*p* < 0.05), as assessed using the least significant difference.

Crude protein is a key metric for evaluating the nutritional quality of HWS, as it reflects the plant’s capacity to assimilate nitrogen and synthesize amino acids and structural proteins [36]. In the control group, CP content was 16.33%, which corresponds to baseline levels observed in unfertilized HWS (Figure 6). The application of calcium nitrate significantly increased CP, peaking at 19.65% in CN3. However, this rise was accompanied by a reduction in biomass, indicating a negative trade-off between nitrogen loading and overall growth. This phenomenon is likely a consequence of metabolic imbalance: excessive nitrate can induce energy-demanding reduction processes, resulting in an accumulation of unused amino acids, increased oxidative stress, and reduced allocation of resources to structural expansion [37].

**Figure 7:**
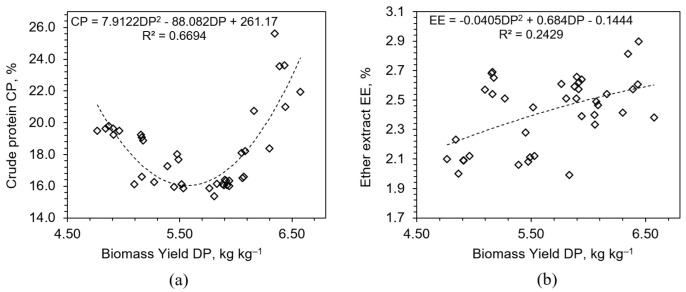
Relationships between biomass yield (DP, kg kg^−1^) and (**a**) crude protein (CP, %) and (**b**) ether extract (EE, %) contents across all fertilizer treatments.

Regression analysis revealed a curvilinear relationship between CP and biomass yield (DP), with CP initially increasing alongside biomass, followed by a plateau and eventual decline at the highest yield levels (Figure 7). This pattern confirms the classical ‘dilution effect’, where the protein concentration decreases due to disproportionate accumulation of water and carbohydrates in rapidly expanding tissues [38]. The mechanism underlying this effect involves carbon–nitrogen metabolic uncoupling under accelerated growth, where structural and storage proteins cannot be synthesized at a rate sufficient to maintain proportional increases in CP.

**Figure 8:**
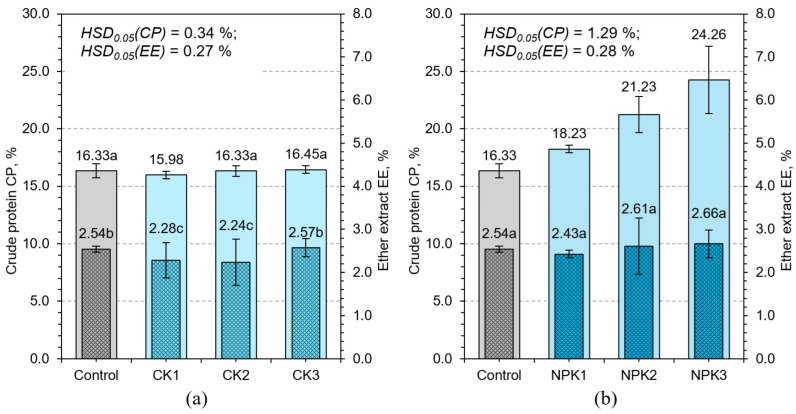
Effect of different potassium sulfate (**a**) and NPK 20–20–20 (**b**) concentrations on crude protein CP and ether extract EE content of HWS. Any two samples with a common letter are not significantly different (*p* < 0.05), as assessed using the least significant difference.

NPK3 exhibited the highest CP content (24.26%) while also supporting maximal biomass production, illustrating that nutrient synergy is essential for the simultaneous optimization of yield and compositional quality (Figure 8). Phosphorus enhances energy metabolism via ATP synthesis, enabling efficient protein assembly and nucleic acid replication [39]. Potassium plays a pivotal role in maintaining turgor pressure, enzyme activation, and regulation of pH and ion gradients, all of which support sustained metabolic throughput and nitrogen assimilation [40].

The intermediate variants CP2 and NPK2 showed high CP levels with moderate biomass, resulting in dense, protein-rich tissues (Figure 6 and Figure 8). These treatments present a valuable compromise for systems where feed quality is prioritized over sheer yield. From a nutritional standpoint, such profiles may improve feed conversion efficiency in livestock by providing concentrated protein per unit of dry matter. These results suggest that CP should be interpreted in context, considering mass and density, rather than as a stand-alone metric. Further physiological insights can be drawn by considering root architecture and nutrient uptake dynamics. It is likely that balanced fertilization not only improved internal biochemical processes but also supported root surface area development and ion transporter activity. Such mechanisms would further enhance nitrogen uptake and assimilation efficiency, explaining the superior CP outcomes in NPK treatments (Figure 8).

Ether extract, representing lipid content, remained relatively stable across treatments (2.07–2.67%), and this lack of variability suggests that lipid metabolism in early vegetative stages of wheat is tightly regulated and not easily influenced by external nutrient availability. Cereals typically synthesize lipids during reproductive phases [41]; therefore, the early-stage hydroponic environment may not provide the developmental triggers necessary for lipid accumulation. This hypothesis is supported by weak correlations between EE and both biomass and dry matter content (R^2^ < 0.25). Despite the low responsiveness, EE remains an important metric for energy value in feed. Its stability ensures a predictable contribution to feed energy balance, which is beneficial for ration formulation. However, any attempts to enhance lipid content through macronutrient fertilization are unlikely to succeed without modifying environmental factors, growth duration, or introducing genetic traits associated with higher baseline lipid production. Strategies such as mild salinity, altered photoperiods, or cultivar selection may offer alternative routes for manipulating EE content.

In conclusion, CP responded dynamically to nutrient composition and concentration, confirming its role as a sensitive and integrative indicator of physiological status under hydroponic conditions. On the other hand, EE remained almost unchanged despite the different fertilizers applied. This indicates that the amount of lipids is biologically inert; it remains stable, as it depends largely on the growth phase and genetics, and not on external fertilizers or environmental conditions. Together, these metrics highlight the importance of understanding both flexible and stable traits in feed optimization.

#### 3.2.2. Crude Fiber and Crude Ash

**Figure 9:**
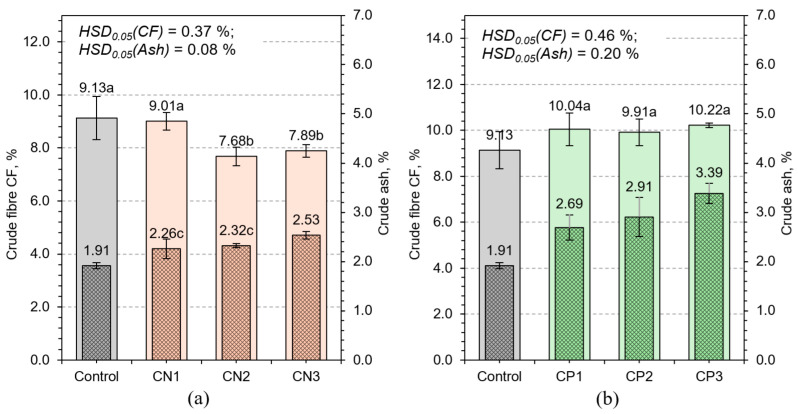
Effect of different calcium nitrate (**a**) and potassium phosphate (**b**) concentrations on crude fiber CF and ash content of HWS. Any two samples with a common letter are not significantly different (*p* < 0.05), as assessed using the least significant difference.

Crude fiber content is indicative of plant tissue maturity and structural development. It encompasses cellulose, hemicellulose, and lignin—compounds that form the primary and secondary cell walls [42]. In the control group, CF was 1.91%, reflecting minimal cell wall thickening typical of early-stage wheat (Figure 9). Fertilization has induced significant increases in CF across all treatments, with marked differences depending on nutrient type. Calcium nitrate treatments resulted in modest CF increases (e.g., CN3–2.53%), reflecting nitrogen’s role in supporting cell elongation but not secondary wall deposition. Nitrogen promotes protein synthesis and cellular expansion, but without adequate phosphorus and potassium, the synthesis of wall polymers remains constrained [43]. This limitation results in tissue that expands volumetrically but lacks commensurate structural reinforcement.

Treatments with elevated phosphorus concentrations, particularly CP3 (3.39%), demonstrated enhanced CF accumulation (Figure 9). Phosphorus is crucial for ATP production, which drives biosynthesis of complex carbohydrates such as hemicellulose and pectin [44]. Additionally, phosphorus supports cell division in meristematic zones, thereby promoting tissue differentiation and wall maturation [45].

**Figure 10:**
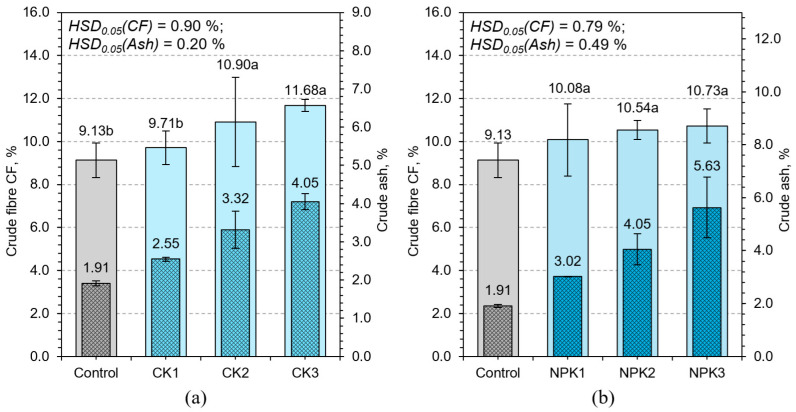
Effect of different potassium sulfate (**a**) and NPK 20–20–20 (**b**) concentrations on crude fiber CF and ash content of HWS. Any two samples with a common letter are not significantly different (*p* < 0.05), as assessed using the least significant difference.

Potassium sulfate further increased the fiber content (CK3–4.05%), highlighting potassium’s regulatory role in carbohydrate partitioning, enzymatic activity, and osmotic balance [46] (Figure 10). The contribution of sulfur may also be significant, as it is involved in the biosynthesis of structural amino acids like cysteine and methionine, which play roles in lignification and cross-linking of wall proteins.

The highest CF value was observed in NPK3 (5.63%), clearly demonstrating the synergistic action of all three macronutrients. This result indicates that only under balanced nutrient supply can the plant simultaneously expand and fortify its tissues. Regression analysis confirmed a strong positive relationship between CF and DM% (R^2^ = 0.81), suggesting that fiber content serves as a reliable proxy for physiological maturity in HWS.

Ash content, representing total inorganic residue post-combustion, is another critical indicator of nutritional value, reflecting mineral accumulation within plant tissues. In the control group, the ash content was 9.13%. CN treatments reduced ash levels (e.g., CN3 = 7.68%), potentially due to ionic imbalance or competitive inhibition of mineral uptake by excess nitrate. In contrast, phosphorus and potassium treatments elevated ash content to over 10%, with the highest values in NPK3 (16.0%). The increase in ash content under NPK and CP treatments likely reflects improved root function, transporter activity, and membrane permeability, all of which enhance mineral uptake. Potassium facilitates the uptake of calcium, magnesium, and micronutrients by stabilizing membrane potential and activating proton pumps. These physiological improvements result in not only greater mineral content but also improved distribution within tissues.

**Figure 11:**
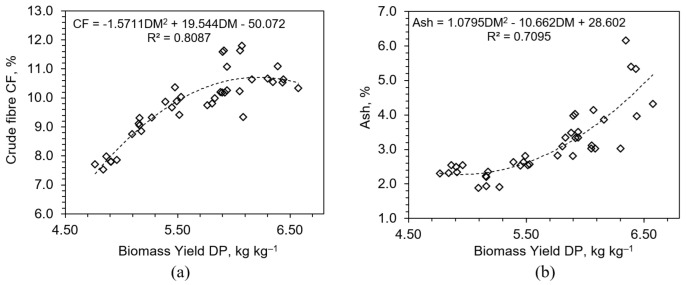
Relationships between biomass yield (DP, kg kg^−1^) and (**a**) crude fiber (CF, %) and (**b**) crude ash (%) contents across all fertilizer treatments.

Regression between ash and DM% revealed a non-linear trend (R^2^ = 0.71), with mineral accumulation plateauing at high DM values (Figure 11). This suggests a physiological ceiling beyond which further mineral uptake is constrained by internal regulatory mechanisms, possibly related to vacuolar storage capacity [47], phloem transport limits [48], or ion toxicity thresholds. Moreover, the concurrent increase in both CF and ash suggests coordinated metabolic prioritization during tissue maturation. As the plant shifts from rapid expansion to strengthening and nutrient loading, structural and mineral constituents are deposited simultaneously. This coordination enhances both mechanical stability and nutritional density of the feed, making such biomass more suitable for animals requiring mineral-rich diets.

In summary, the CF and ash content are both highly responsive to fertilization, particularly under balanced nutrient regimes. They offer complementary insights into the structural and mineral maturation of HWS. Their correlations with DM% and distinct patterns across treatments underscore their value as diagnostic indicators for both physiological assessment and quality control in precision agriculture systems.

### 3.3. Practical Implications for Fertilization Strategy

The comprehensive analysis of biomass yield, dry matter accumulation, and feed quality parameters under varying fertilization regimes reveals insights for optimizing HWS production. The findings indicate that no single macronutrient guarantees both high biomass and nutritional quality. Instead, optimal outcomes are achieved through the combination and concentration of nitrogen, phosphorus, and potassium.

For producers aiming to maximize biomass yield, the use of balanced NPK fertilization, particularly at moderate to high concentrations (e.g., NPK2 and NPK3) is recommended. In contrast, if the production goal is to enhance nutrient density, especially crude protein or mineral content, phosphorus-dominant regimes (e.g., CP2 or CP3) offer a valuable alternative. These variants achieved significant improvements in CP, CF, and ash content while avoiding the dilution effect commonly seen in high-yield systems. Also, the low responsiveness of the ether extract to fertilization implies that strategies aimed at increasing lipid content should involve alternative approaches, such as cultivar selection, modification of growth duration, or controlled stress induction, rather than nutrient manipulation. Importantly, the strong regression patterns observed between dry matter and both CF and ash suggest that simple indirect measurements (e.g., DM%) can serve as reliable indicators of HWS quality traits. This opens the possibility for streamlined monitoring protocols in commercial systems.

It is worth noting that the biochemical composition of the hydroponic wheat sprouts obtained in this study suggests specific applications for different livestock categories. From a nutritional standpoint, the enhanced crude protein (up to 24.26%) and ash content (up to 16.0%) observed under NPK3 treatment suggest that hydroponic wheat sprouts can serve as a protein- and mineral-rich supplement in ruminant diets, potentially reducing the need for additional concentrates or mineral premixes. Moreover, the relatively low crude fiber content ensures good digestibility, making the sprouts suitable not only for cattle and sheep but also potentially for monogastric animals when included in balanced rations. Compared to conventional forages like alfalfa (~18–20% CP) or maize silage (~8–10% CP), the protein concentration in HWS under optimized fertilization is highly competitive. These compositional traits highlight the potential of HWS as a high-value, space- and resource-efficient feed component in precision livestock nutrition systems.

### 3.4. Limitations of the Study

While the study presents robust evidence on the influence of macronutrient composition on biomass and feed quality traits in HWS, certain limitations should be acknowledged. Firstly, the short cultivation period (7 days) may have constrained the full expression of slower-developing traits, particularly those related to lipid accumulation and secondary metabolite pathways. Longer growth durations might reveal additional nutrient effects or temporal shifts in compositional traits such as ether extract or complex structural carbohydrates.

Secondly, the ash content was assessed as a total inorganic residue, without elemental differentiation. As such, the specific contributions of individual macro- and micronutrients (e.g., calcium, magnesium, iron, and zinc) remain unresolved. Future studies using inductively coupled plasma optical emission spectroscopy (ICP-OES) or similar elemental analysis techniques could clarify the precise mineral dynamics and potential antagonisms or synergies among elements.

Thirdly, this study was conducted under highly controlled conditions in a growth chamber, which, while eliminating environmental noise, limits the extrapolation of findings to commercial-scale systems that involve variable humidity, temperature, and light quality. Additionally, potential cultivar-specific responses were not addressed, as only one wheat genotype was tested. Genetic variation may influence nutrient responsiveness, and incorporating multiple cultivars would enhance the generalizability of findings. Despite these limitations, the study provides a solid foundation for further research and optimization in controlled environment HWS systems.

## 4. Conclusions

This study demonstrated that both the type and concentration of macronutrient fertilizers significantly influence the biomass yield and nutritional composition of HWS. The highest biomass yield (6.39 kg kg^−1^ DP) was achieved under NPK3 treatment, which also resulted in the highest crude protein (24.26%) and crude fiber (5.63%) content, indicating that balanced fertilization with nitrogen, phosphorus, and potassium promotes both productivity and feed quality. Excessive calcium nitrate (CN3) led to a reduced yield (4.84 kg kg^−1^ DP) despite elevated protein levels (19.65%), highlighting a trade-off between nitrogen-induced protein synthesis and overall vegetative growth. Potassium phosphate treatments, particularly CP2 and CP3, improved both protein (up to 17.65%) and ash content (10.22%) without causing yield suppression, suggesting these are effective strategies for enhancing nutrient density. Regression analyses revealed strong correlations between biomass and dry matter (R^2^ = 0.86), and between dry matter and both fiber (R^2^ = 0.81) and ash content (R^2^ = 0.71), indicating these parameters are physiologically linked and can be used for predictive quality assessments. The relatively unchanged ether extract content (2.07–2.67%) across all treatments underscores its developmental regulation and limited responsiveness to macronutrient manipulation. Among all treatments, NPK3 demonstrated the best overall performance, achieving the highest biomass yield and superior nutritional quality, making it the most suitable fertilization regime for hydroponic wheat sprout production.

This study contributes a comprehensive perspective by integrating statistical correlations with mechanistic physiological interpretations, providing new insights into nutrient use efficiency and compositional optimization. It also demonstrates how regression-based modeling can help predict critical quality traits, enabling data-driven decisions in modern HWS production systems. Collectively, the findings underscore the importance of nutrient synergy and targeted fertilization strategies to optimize both yield and compositional quality in controlled environment HWS systems, providing practical guidance for sustainable feed production in precision agriculture.

## Figures and Tables

**Table 1:** Dynamic parameters of empirical research.

	Temperature, °C	Humidity, %	CO_2_, ppm	VPD *
Control	21.87 ± 0.05	37.88 ± 0.26	591.58 ± 2.26	1.616 ± 0.008
CN1	21.69 ± 0.05	47.56 ± 0.24	605.96 ± 2.54	1.349 ± 0.008
CN2	21.26 ± 0.05	44.2 ± 0.21	601.91 ± 2.68	1.396 ± 0.007
CN3	20.95 ± 0.05	34.84 ± 0.38	616.35 ± 3.02	1.602 ± 0.011
CP1	20.49 ± 0.05	45.73 ± 0.59	609.26 ± 2.78	1.31 ± 0.005
CP2	21.18 ± 0.05	48.78 ± 0.52	642.97 ± 2.77	1.29 ± 0.008
CP3	20.67 ± 0.05	49.78 ± 0.26	636.68 ± 3.78	1.23 ± 0.006
CK1	21.13 ± 0.05	41.82 ± 0.35	589.69 ± 3.01	1.46 ± 0.006
CK2	21.14 ± 0.05	47.62 ± 0.62	630.42 ± 2.12	1.31 ± 0.007
CK3	21.47 ± 0.05	42.62 ± 0.57	633.76 ± 3.59	1.47 ± 0.008
NPK1	20.57 ± 0.05	56.96 ± 0.28	624.91 ± 3.16	1.04 ± 0.008
NPK2	20.46 ± 0.05	40.76 ± 0.71	648.26 ± 3.43	1.43 ± 0.008
NPK3	21.18 ± 0.05	43.86 ± 0.42	591.43 ± 2.23	1.41 ± 0.005

* Vapor pressure deficit.

**Table 2:** Nutrient concentrations in solution and physicochemical properties of fertilizer treatments.

		Nutrients Concentration in Solution, mg L^–1^			EC	pH
	Control *	–	–		0.643 ± 0.015	5.8–6.0
		N (NO_3^−^_)	Ca^2+^			
Ca(NO_3_)^2^	CN1	100	143		1.316 ± 0.061	5.8–6.0
	CN2	200	286		2.070 ± 0.220	5.8–6.0
	CN3	300	429		2.928 ± 0.086	5.8–6.0
		P (H_2_PO_4^−^_)	K^+^			
KH_2_PO_4_	CP1	100	126		0.782 ± 0.029	5.8–6.0
	CP2	200	252		0.913 ± 0.059	5.8–6.0
	CP3	300	378		1.045 ± 0.088	5.8–6.0
		K^+^	S (SO_4^2−^_)			
K_2_SO_4_	CK1	100	18		0.940 ± 0.060	5.8–6.0
	CK2	200	36		1.228 ± 0.120	5.8–6.0
	CK3	300	54		1.518 ± 0.175	5.8–6.0
NPK 20-20-20		N (NO_3^−^_, NH_4^+^_, NH_2_)	P (H_2_PO_4^−^_)	K^+^		
	NPK1	100	43.6	83	1.600 ± 0.124	5.8–6.0
	NPK2	200	87.2	166	2.551 ± 0.248	5.8–6.0
	NPK3	300	130.8	249	3.489 ± 0.371	5.8–6.0

* Tap water.

## Data Availability

The original contributions presented in this study are included in the article. Further inquiries can be directed to the corresponding author.
